# Brain Frontal-Lobe Misery Perfusion in COVID-19 ICU Survivors: An MRI Pilot Study

**DOI:** 10.3390/brainsci14010094

**Published:** 2024-01-18

**Authors:** Jie Song, Shivalika Khanduja, Hannah Rando, Wen Shi, Kaisha Hazel, George Paul Pottanat, Ebony Jones, Cuimei Xu, Zhiyi Hu, Doris Lin, Sevil Yasar, Hanzhang Lu, Sung-Min Cho, Dengrong Jiang

**Affiliations:** 1 Department of Biomedical Engineering, Johns Hopkins University School of Engineering, Baltimore, MD 21218, USA; 2 Department of Surgery, Johns Hopkins University School of Medicine, Baltimore, MD 21287, USA; 3 Department of Biomedical Engineering, Johns Hopkins University School of Medicine, Baltimore, MD 21287, USA; 4 The Russell H. Morgan Department of Radiology & Radiological Science, Johns Hopkins University School of Medicine, 600 N. Wolfe Street, Park 324, Baltimore, MD 21287, USA; 5 Department of Medicine, Johns Hopkins University School of Medicine, Baltimore, MD 21287, USA; 6 F. M. Kirby Research Center for Functional Brain Imaging, Kennedy Krieger Research Institute, Baltimore, MD 21205, USA; 7 Department of Neurology, Neurosurgery, Surgery, Anesthesiology, and Critical Care Medicine, Johns Hopkins University School of Medicine, Baltimore, MD 21287, USA

**Keywords:** COVID-19, ICU, post-acute COVID-19 syndrome, oxygen extraction fraction, cerebral blood flow, misery perfusion, cerebral microvascular damage

## Abstract

Post-acute COVID-19 syndrome (PCS) is highly prevalent. Critically ill patients requiring intensive care unit (ICU) admission are at a higher risk of developing PCS. The mechanisms underlying PCS are still under investigation and may involve microvascular damage in the brain. Cerebral misery perfusion, characterized by reduced cerebral blood flow (CBF) and elevated oxygen extraction fraction (OEF) in affected brain areas, has been demonstrated in cerebrovascular diseases such as carotid occlusion and stroke. This pilot study aimed to examine whether COVID-19 ICU survivors exhibited regional misery perfusion, indicating cerebral microvascular damage. In total, 7 COVID-19 ICU survivors (4 female, 20–77 years old) and 19 age- and sex-matched healthy controls (12 female, 22–77 years old) were studied. The average interval between ICU admission and the MRI scan was 118.6 ± 30.3 days. The regional OEF was measured using a recently developed technique, accelerated T_2_-relaxation-under-phase-contrast MRI, while the regional CBF was assessed using pseudo-continuous arterial spin labeling. COVID-19 ICU survivors exhibited elevated OEF (β = 5.21 ± 2.48%, *p* = 0.047) and reduced relative CBF (β = −0.083 ± 0.025, *p* = 0.003) in the frontal lobe compared to healthy controls. In conclusion, misery perfusion was observed in the frontal lobe of COVID-19 ICU survivors, suggesting microvascular damage in this critical brain area for high-level cognitive functions that are known to manifest deficits in PCS. Physiological biomarkers such as OEF and CBF may provide new tools to improve the understanding and treatment of PCS.

## 1. Introduction

Coronavirus disease 2019 (COVID-19), caused by the infection of severe acute respiratory syndrome coronavirus 2 (SARS-CoV-2), has presented unprecedented challenges to global healthcare systems. Although the mortality rate of COVID-19 has significantly decreased with the evolution of SARS-CoV-2 and advances in COVID-19 vaccines and treatments, post-acute COVID-19 syndrome (PCS) is highly prevalent and has garnered increasing attention [1]. PCS is characterized by signs and symptoms that develop during and after SARS-CoV-2 infection and persist for over 12 weeks [2]. It is estimated that approximately 40% of patients diagnosed with COVID-19 suffer from neurological and neuropsychiatric symptoms of PCS, including fatigue, brain fog, sleep disturbances, memory issues, etc. [3]. Notably, reports indicate that COVID-19 intensive care unit (ICU) survivors, namely patients who were critically ill due to COVID-19 and required ICU admission, are at a higher risk of developing long-term neurological deficits and cognitive impairments than individuals with mild symptoms [4]. The mechanisms underlying PCS are still under investigation and have been suggested to involve cerebral microvascular damage among some proposed mechanisms [1,5,6]. Cerebral microvascular damage may disrupt oxygen delivery and utilization in the brain, leading to cognitive dysfunctions such as brain fog.

Misery perfusion, characterized by elevated oxygen extraction fraction (OEF) and diminished cerebral blood flow (CBF) in affected brain areas, has been demonstrated as a reliable hallmark in cerebrovascular diseases. For example, in chronic major cerebral artery occlusive diseases, misery perfusion in the hemisphere ipsilateral to the occlusion is a strong and independent predictor of future ischemic stroke risk [7,8]. In ischemic stroke, misery perfusion signifies salvageable penumbra in the hyperacute to the acute stage [9,10,11]. In cerebral small vessel diseases, misery perfusion has been observed in the centrum semiovale among lacunar stroke patients with severe white-matter lesions [12].

Therefore, we hypothesize that COVID-19 ICU survivors, who are among the most severe cases of COVID-19, will exhibit long-term misery perfusion in certain brain regions, indicating cerebral microvascular damage. In this pilot study, we utilized a recently developed MRI technique, the accelerated T_2_-relaxation-under-phase-contrast (aTRUPC) method [13], to evaluate the regional OEF and employed pseudo-continuous arterial spin labeling (pCASL) [14,15,16] to assess the regional CBF in COVID-19 ICU survivors, in comparison with healthy controls.

## 2. Materials and Methods

### 2.1. Participants

The study protocol was approved by the Johns Hopkins University Institutional Review Board (IRB00276578, approved on 19 May 2021 and IRB00048412, approved on 18 October 2021). Each subject provided written informed consent before participating in this study.

COVID-19 ICU survivors were enrolled between October 2021 and January 2023. The inclusion criteria were age > 18 years and a history of ICU admission due to COVID-19. The exclusion criteria included a history of diagnosed cognitive impairment and contraindications for MRI.

Healthy adults were recruited between April 2022 and July 2023 to serve as the control group. The inclusion criteria comprised general good health based on self-report, no history of hospitalization related to COVID-19, and the absence of COVID-19 symptoms or positive test results for COVID-19 at the time of the MRI scan. Subjects were excluded if they had cognitive impairment or contraindications for MRI.

### 2.2. MRI Experiments

All participants were scanned on a 3T Siemens Prisma scanner (Siemens Healthineers, Erlangen, Germany), using a 32-channel head coil for receiving and the body coil for transmission.

aTRUPC MRI was utilized to measure the regional OEF in each subject. Briefly, aTRUPC uses phase-contrast complex subtraction to isolate pure blood signal in major cerebral veins and employs T_2_ preparation with varying effective echo times (eTEs) to quantify the blood T_2_, which can be converted to venous oxygenation (Y_v_) through a calibration model [13,17]. Subsequently, OEF can be calculated as the difference between Y_v_ and arterial oxygenation (Y_a_), which was measured with a pulse oximeter on the subject’s finger outside of the MRI scanner room. In this study, aTRUPC had the following parameters [13]: 2D single-slice in the mid-sagittal plane, field of view (FOV) = 200 × 200 mm^2^, slice thickness = 10 mm, reconstructed in-plane resolution = 0.8 × 0.8 mm^2^, 3 eTEs for T_2_ preparation (0, 40 and 80 ms), velocity encoding = 15 cm/s, GRAPPA factor = 2, 4 averages, and total scan time = 2.1 min.

Each subject also underwent a pCASL scan to assess the regional CBF, using the following parameters [14]: labeling duration = 1800 ms, post-labeling delay = 2200 ms, FoV = 220 × 220 × 144 mm^3^, voxel size = 3.4 × 3.4 × 4.0 mm^3^, repetition time (TR) = 4320 ms, echo time = 12.6 ms, and scan time = 4.7 min. For quantification purposes, an M_0_ scan was acquired with TR = 10,000 ms and scan duration = 1.5 min.

In addition, 3D T_1_-weighted magnetization-prepared rapid gradient echo (MPRAGE) was acquired for brain parcellation, using the following parameters: FoV = 256 × 256 × 176 mm^3^, voxel size = 1.0 × 1.0 × 1.0 mm^3^, TR = 2530 ms, inversion time = 1100 ms, and scan time = 6 min.

### 2.3. aTRUPC Data Processing

**Figure 1:**
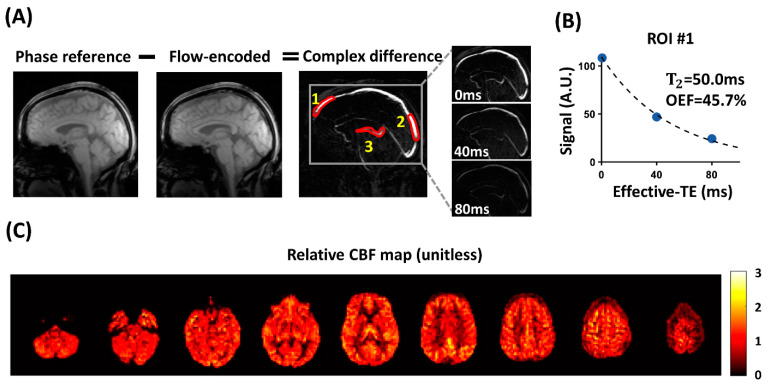
Representative aTRUPC and pCASL data of a COVID-19 ICU survivor: (**A**) aTRUPC uses phase-contrast complex subtraction between phase-reference and flow-encoded images to yield complex difference (CD) images, in which the static tissues are canceled out, leaving only the blood signals in the vessels. Zoom-in views of the CD images are presented for all eTEs. The red contours on the CD image delineate the ROIs for OEF quantification. (**B**) Scatterplot showing venous signal of ROI #1 as a function of effective TEs. Fitted venous blood T_2_ and the corresponding OEF values are also shown. (**C**) The relative CBF map plotted using pCASL in this patient. Abbreviations: aTRUPC: accelerated T_2_ relaxation under phase contrast; pCASL: pseudo-continuous arterial spin labeling; COVID-19: coronavirus disease 2019; ICU: intensive care unit; CD: complex difference; eTE: effective echo time; ROI: regions of interest; OEF: oxygen extraction fraction; CBF: cerebral blood flow.

The aTRUPC data were processed to derive Y_v_ values in major cerebral veins [13,18] using in-house MATLAB (R2022b, MathWorks, Natick, MA, USA) scripts. The Statistical Parametric Mapping tool (SPM12, release date: 13th January 2020, University College London, London, UK) was utilized for motion correction across images. Complex subtraction was conducted between phase-reference images and flow-encoded images to generate complex difference (CD) images (Figure 1A).

#### 2.3.1. Iterative Maxwell and Eddy-Current Correction 

Phase errors (Δε) induced by Maxwell concomitant field and eddy-current effects result in residual tissue signals in the CD images, which may introduce bias into the T_2_ quantification and thus need to be corrected [13]. When there are no Maxwell concomitant field or eddy-current effects, the phase difference (Δφ) between the phase-reference and flow-encoded images should be zero for static tissue voxels. Therefore, Δε can be estimated from the measured Δφ of static tissue voxels. To automatically compute a static tissue mask and perform the phase correction, we employed the following iterative algorithm:

Step 1: A brain mask was derived from the phase-reference image, and an initial vessel mask was obtained from the CD image at eTE = 0 ms, both using Otsu’s method [19]. Note that this initial vessel mask may contain non-vessel voxels due to residual tissue signals in the CD image.

Step 2: A static tissue mask was generated by subtracting the vessel mask from the brain mask. Δφ within the static tissue mask was fitted to a hyperplane [13,20]:

$$\Delta \varphi =c_{1}+c_{2}X+c_{3}Y+c_{4}XY+c_{5}X^{2}+c_{6}Y^{2}$$

Step 3: The phase error Δε was estimated for each voxel as the corresponding values on the fitted hyperplane. Subsequently, Δε was removed from the complex subtraction procedure, resulting in corrected CD images:

$$CD_{corrected}=I_{phase-reference}\cdot e^{-i\Delta \varepsilon }-I_{flow-encoded}$$

As the residual tissue signals were largely eliminated in CD_corrected_, we recomputed the vessel mask using CD_corrected_ and repeated Step 2 and Step 3. Based on our experience, five iterations were sufficient to achieve convergence for this iterative algorithm.

#### 2.3.2. Semi-Automatic ROI Analysis

For quantification purposes, three regions of interest (ROIs) were determined using a semi-automatic algorithm: ROI #1 and #2 were positioned near the frontal and posterior ends of the superior sagittal sinus (SSS), respectively, while ROI #3 included the internal cerebral veins (ICVs). The locations of the three ROIs are illustrated in Figure 1.

To minimize rater dependence in selecting the ROIs, we developed a semi-automatic algorithm. Initially, a preliminary vessel mask was manually drawn on CD_corrected_ at eTE = 0 ms to encompass major cerebral veins, including the SSS, straight sinus, the vein of Galen (GV), and ICVs. The final vessel mask was obtained by applying a threshold within this preliminary vessel mask [13].

**Figure 2:**
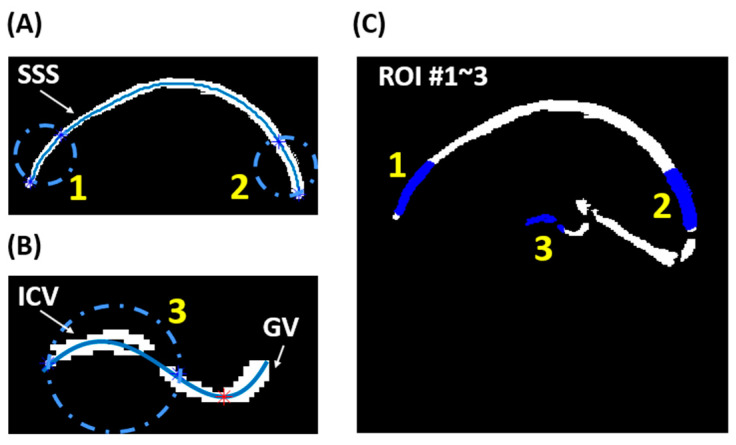
Semi-automatic ROI delineation: (**A**) ROI selection on the SSS; (**B**) ROI selection for ICV; (**C**) locations of the three ROIs on the final vessel mask.

For ROI #1 and #2, we extracted the skeleton of the SSS segment of the final vessel mask, as shown in Figure 2A. The anteriormost and posteriormost 30 mm segments along the SSS skeleton were selected as ROI #1 and #2, respectively.

For ROI #3, we extracted the skeleton of the ICV and GV segments from the final vessel mask, as depicted in Figure 2B. To distinguish the ICV from GV, we smoothed the skeleton using polynomial curve fitting and identified the lowest point of the smoothed curve (red asterisk in Figure 2B). ROI #3 was selected as the skeleton segment located 10 mm anterior to the red asterisk. Figure 2C presents an overlay of the final three ROIs on the final vessel mask.

Signals within each ROI were spatially averaged and fitted as a monoexponential function of eTEs to yield the blood T_2_, which was subsequently translated to Y_v_ using a published calibration model that has been validated against the gold-standard ^15^O-PET method [17,21]. The T_2_ − Y_v_ conversion factored in the hematocrit level, which was assumed to be 0.4 for females and 0.42 for males. Subsequently, the OEF of each ROI was calculated as OEF = (Y_a_ − Y_v_)/Y_a_ × 100%, where Y_a_ represents the arterial oxygenation measured with a pulse oximeter on the subject’s finger outside of the MRI scanner room.

### 2.4. pCASL Data Processing

The pCASL data were processed using the open access ASL-MRICloud tool (Version 5) [22] to obtain voxel-wise maps of CBF. To pair with aTRUPC regional OEF measurements, we derived the average CBF values from the three selected ROIs based on T_1_-MPRAGE segmentations using the MRICloud platform [23]. According to the general venous drainage territories, the frontal SSS mainly drains the frontal gray matter (GM) and the posterior SSS receives drainage from most of the neocortical GM, whereas the ICVs mainly drain the basal ganglia and thalami (BGT) [24]. Therefore, to pair with the OEF ROIs, CBF ROI #1 covered the frontal GM, CBF ROI #2 included the GM of all brain lobes, and CBF ROI #3 covered the BGT. It is known that the quantification of absolute CBF in pCASL can be influenced by several methodological factors such as variations in labeling efficiency, arterial transit time, and blood/tissue T_1_ [14,25,26]. Therefore, this study focused on the comparison of relative CBF (rCBF) values that are regional CBF values normalized using whole-brain averaged CBF.

### 2.5. Statistical Analysis

All statistical analyses were conducted using MATLAB (R2022b). The Shapiro–Wilk test was employed to assess the normality of the data. Data with a normal distribution were presented as mean ± standard deviation, whereas data not following a normal distribution were presented as median and range. A two-sample *t*-test, Wilcoxon rank-sum test, or Fisher’s exact test was utilized to compare the characteristics between COVID-19 ICU survivors and control subjects, as deemed appropriate.

Linear regression analyses were employed to compare the OEF and rCBF values of each ROI between the COVID-19 ICU survivors and the control group, with age as a covariate. We also repeated the regression analyses after adding sex as an additional covariate.

In all analyses, a two-tailed *p*-value < 0.05 was considered statistically significant.

## 3. Results

Table 1 lists the characteristics of COVID-19 ICU survivors and healthy controls, showing no significant differences in sex (*p* = 1.00), age (*p* = 0.71), or Y_a_ (*p* = 0.72) between the two groups. Regarding COVID-19 ICU survivors, the median time from symptom onset to ICU admission was 1 day (ranging from 1 to 8 days), with an average ICU stay of 20.0 ± 18.2 days and an average length of hospital stay of 29.1 ± 25.1 days. Two patients were intubated during their ICU stay. The average interval between ICU admission and the MRI scan was 118.6 ± 30.3 days. A subset of patients reported post-acute COVID-19 symptoms, including coughing (*N* = 3), shortness of breath (*N* = 4), and fatigue (*N* = 3).

Figure 1 shows representative aTRUPC OEF and pCASL CBF data of a COVID-19 ICU survivor (female, 56 years old). Table 2 lists the ROI OEF and rCBF values of COVID-19 ICU survivors and healthy controls.

Table 3 summarizes the results of the linear regression analyses. We found that COVID-19 ICU survivors had significantly elevated OEF in the frontal SSS (*p* = 0.047) and reduced rCBF in the frontal GM (*p* = 0.003). No significant differences in OEF or rCBF were found in other ROIs (*p* > 0.2). In addition, OEF in the frontal SSS increased with age (β = 0.11 ± 0.05%/year, *p* = 0.03). rCBF in the frontal GM decreased with age (β = −0.0018 ± 0.00051/year, *p* = 0.002), while rCBF in BGT increased with age (β = 0.0031 ± 0.00064/year, *p* = 0.0001). We repeated the regression analyses after adding sex as an additional covariate. We found that sex did not have a significant effect (*p* > 0.1), and the findings concerning OEF and CBF remained consistent (Supplemental Material Table S1), although the group difference in OEF in the frontal SSS became marginally significant (*p* = 0.054).

## 4. Discussion

In this pilot study, we observed that, compared to healthy controls, COVID-19 ICU survivors exhibited significantly increased OEF and decreased rCBF in the frontal lobe, consistent with the “misery perfusion” pattern.

The frontal lobe, including the prefrontal cortex, plays a critical role in high-level cognitive functions such as memory, attention, and executive function [27]. Studies investigating the neurocognitive sequelae of COVID-19 have reported impaired memory, attention, language, and executive functions among post-infection patients [28,29,30]. Specifically, in hospitalized COVID-19 patients, the most severely affected cognitive domains appear to be memory and executive function [30]. In our study, we observed misery perfusion in the frontal lobe in COVID-19 ICU survivors with an average interval of 118.6 ± 30.3 days after ICU admission. This finding suggests persistent microvascular damage in the frontal lobe of these patients, which may disrupt the delivery of oxygen and nutrients to brain tissues, thereby contributing to deficits in cognitive functions.

Cerebral microvascular damage associated with COVID-19 has been well documented in both post-mortem and in vivo studies [5,31,32,33], yet the underlying mechanisms remain unclear. For example, in a recent autopsy study examining the brains of patients who died of COVID-19, Lee et al. demonstrated multifocal vascular damage characterized by the leakage of serum proteins into the brain parenchyma, accompanied by widespread endothelial cell activation [5]. An in vivo MRI study also suggested blood–brain barrier breakdown in COVID-19 ICU survivors [34]. It is worth noting that autopsy studies have either found no presence of the SARS-CoV-2 virus in the brain or detected it at very low levels, suggesting that the neurovascular injury is more likely a result of cytotoxicity targeting endothelial cells induced by antibodies or cytokines, rather than the direct SARS-CoV-2 infection of the endothelium [5,35,36].

Several studies have reported post-acute physiological or metabolic abnormalities in the frontal lobe or other brain regions among patients following COVID-19 infections. Research utilizing 18-fluorodeoxyglucose positron emission tomography (^18^F-FDG PET) has identified impaired glucose metabolism in various brain regions, including the frontal lobe, temporal lobe, thalamus, brainstem, and cerebellum in patients with PCS [30,37,38]. Investigations employing pCASL have revealed hypoperfusion in the frontal lobe [39,40], temporal lobe [40], subcortical nuclei, and limbic structures [41] among post-COVID-19 patients. A recent study showed increased global OEF in PCS patients compared to healthy controls, with higher OEF associated with slower gait speed [42]. To the best of our knowledge, our study is the first to investigate post-acute alterations in the regional OEF among patients following COVID-19 infections. The elevated OEF we observed in the frontal lobe likely represents a compensatory response to reduced blood flow.

In addition, we found significant age effects on OEF and rCBF in this study (Table 3). The observed positive association between age and OEF in the frontal SSS aligned with several previous studies indicating an increase in OEF with age [18,43,44,45]. Regarding CBF, it has been consistently reported that the CBF of cortical GM decreased with age [46,47,48]. The literature on subcortical GM yields mixed results. While some studies report insignificant age-related changes in the CBF of subcortical GM [48], others show an increase in the CBF of subcortical GM with age [49,50]. In our study, we observed a negative association between age and rCBF in the frontal GM, contrasting with a positive association between age and rCBF in the BGT. Since rCBF was computed as the ratio of ROI CBF to whole-brain averaged CBF, our results indicated that the rate of age-related CBF decline is faster in the frontal GM compared to the whole-brain average, while the decrease rate is slower (or possibly absent) in the BGT. These findings suggest that age effects on brain physiology vary across different brain regions.

The conventional gold standard for measuring OEF and CBF in humans is PET with ^15^O-labeled radiotracers [51,52]. However, the broad clinical applications of this ^15^O-PET method have been impeded by radiation exposure, complex logistics, and the need for an on-site cyclotron to produce the ^15^O isotope, which has a short half-life of approximately 2 min [51,52,53]. In this pilot study, we utilized aTRUPC MRI to evaluate the regional OEF and employed pCASL to measure the regional CBF. The accuracy of aTRUPC in OEF measurement has been validated against gold-standard blood gas oximetry [54]. Several studies have revealed significant correlations between pCASL and ^15^O-PET in CBF measurement, although bias in absolute CBF values may exist [55,56]. Since both aTRUPC and pCASL are non-invasive; do not involve any ionizing radiation, nor do they require any contrast agents; and can be performed on standard clinical MRI scanners, they have great potential for broad clinical applications. Therefore, the findings of this work may be easily translated to other studies.

Our study had several limitations. Firstly, the sample size of COVID-19 ICU survivors was small. Therefore, this study should be viewed as a pilot study, and our findings are intended to generate hypotheses for future large-scale studies in PCS patients.

Secondly, we did not conduct standardized cognitive tests on all participants because the primary goal of this pilot study was to assess whether COVID-19 ICU survivors exhibited alterations in brain perfusion and oxygen extraction. Consequently, we were unable to examine the associations of OEF or CBF measures with cognitive dysfunctions, which are known to frequently occur in PCS patients [28,29,30]. Several ^18^F-FDG PET studies have reported associations between cerebral glucose hypometabolism and cognitive dysfunctions in PCS patients. For example, Hosp et al. found that the expression of a frontoparietal hypometabolism pattern was associated with impaired cognition evaluated using the Montreal Cognitive Assessment [30]. Guedj et al. reported that the decreased metabolism of the brainstem and cerebellum was correlated with an increased number of functional complaints [38]. On the other hand, Blazhenets et al. showed that the reversal of the frontoparietal hypometabolism pattern was accompanied by a significant improvement in cognition [57]. Since the brain’s glucose metabolism is closely related to its oxygen metabolism [58], which is determined by brain perfusion and oxygen extraction, it is possible that the OEF and CBF measures may be correlated with patients’ cognitive performance and may be useful in monitoring the progression of PCS or patients’ recovery from COVID-19. Evaluating such correlations will be the goal of our future studies.

Thirdly, it has been reported that ICU admission itself may lead to long-lasting complications in ICU survivors, known as post-ICU syndrome [59,60]. Since our control group did not experience ICU admission, it was not possible for us to differentiate COVID-19-related effects from those associated with post-ICU syndrome. Future studies should involve subjects with a history of ICU admission due to non-COVID diseases.

## 5. Conclusions

This pilot study provided preliminary evidence indicating that COVID-19 ICU survivors exhibited misery perfusion in the frontal lobe. Physiological parameters such as OEF and CBF may offer new tools to improve our understanding of long-term alterations in brain physiology and function after severe COVID-19 infection.

## Figures and Tables

**Table 1:** Demographical and clinical characteristics of the participants.

	COVID-19 ICU Survivors	Control	*p*-Value
*N*	7	19	N/A
Sex (Female) *	4 (57.1%)	12 (63.2%)	1.00 ^a^
Age (years) ^†^	50 (20–77)	64 (22–77)	0.71 ^b^
Y_a_ (%) ^‡^	97.7 ± 1.5	97.5 ± 1.0	0.72 ^c^
Length of hospital stay ^‡^	29.1 ± 25.1	NA	NA
Time from symptom onset to ICU admission (days) ^†^	1 (1–8)	NA	NA
Duration of stay in ICU (days) ^‡^	20.2 ± 18.2	NA	NA
Time from ICU admission to MRI (days) ^‡^	118.6 ± 30.3	NA	NA
Intubation *	2 (28.6%)	NA	NA
Post-acute COVID-19 symptoms			
Coughing *	3 (42.9%)	NA	NA
Shortness of breath *	4 (57.1%)	NA	NA
Fatigue *	3 (42.9%)	NA	NA

* Data are numbers of participants, followed by percentages in parentheses. ^†^ Data are medians, with ranges in parentheses. ^‡^ Data are means ± standard deviations. ^a^ Fisher’s exact test. ^b^ Wilcoxon rank-sum test. ^c^ Two-sample *t*-test. Abbreviations: COVID-19: coronavirus disease 2019; ICU: intensive care unit; Y_a_: arterial oxygenation.

**Table 2:** Regional OEF and rCBF values of COVID-19 ICU survivors and healthy controls.

Variable	ROI	COVID-19 ICU Survivors	Control
OEF (%)	frontal SSS	38.3 ± 9.0	33.5 ± 4.7
	posterior SSS	38.2 ± 8.4	35.4 ± 5.1
	ICV	32.5 ± 11.5	27.9 ± 7.7
rCBF (unitless)	frontal GM	1.20 ± 0.07	1.27 ± 0.07
	GM of all lobes	1.22 ± 0.04	1.23 ± 0.03
	BGT	0.96 ± 0.15	0.95 ± 0.08

Data are means ± standard deviation. Abbreviations: OEF: oxygen extraction fraction; rCBF: relative cerebral blood flow; COVID-19: coronavirus disease 2019; ICU: intensive care unit; SSS: superior sagittal sinus; ICV: internal cerebral veins; GM: gray matter; BGT: basal ganglia and thalami.

**Table 3:** Results of linear regression analyses for regional OEF and rCBF.

Model	Dependent Variable	Independent Variables	Coefficient ± Standard Error	*p*-Value
OEF1	OEF in frontal SSS	Group	5.21 ± 2.48%	0.047
		Age	0.11 ± 0.050%/year	0.03
OEF2	OEF in posterior SSS	Group	3.06 ± 2.65%	0.26
		Age	0.078 ± 0.053%/year	0.16
OEF3	OEF in ICV	Group	4.90 ± 3.93%	0.23
		Age	0.068 ± 0.079%/year	0.40
rCBF1	rCBF in frontal GM	Group	−0.083 ± 0.025	0.003
		Age	−0.0018 ± 0.00051/year	0.002
rCBF2	rCBF in GM of all lobes	Group	−0.018 ± 0.015	0.24
		Age	−0.00056 ± 0.00030/year	0.08
rCBF3	rCBF in BGT	Group	0.027 ± 0.032	0.41
		Age	0.0031 ± 0.00064/year	0.0001

Group = 1 for COVID-19 ICU survivors and Group = 0 for healthy controls. Abbreviations: OEF: oxygen extraction fraction; rCBF: relative cerebral blood flow; SSS: superior sagittal sinus; ICV: internal cerebral veins; GM: gray matter; BGT: basal ganglia and thalami; COVID-19: coronavirus disease 2019; ICU: intensive care unit.

## Data Availability

Data will be made available on request due to privacy restrictions. The source code of this work is available at: https://github.com/NeuroFunctionLab/aTRUPC_processing (accessed on 16 December 2023).

## Supplementary Materials

The following supporting information can be downloaded at: https://www.mdpi.com/article/10.3390/brainsci14010094/s1, Table S1. Results of linear regression analyses for regional OEF and rCBF, with sex as an additional covariate.
