# Supplementary material for: Brain Frontal-Lobe Misery Perfusion in COVID-19 ICU Survivors: An MRI Pilot Study
Source: Brain Sci. 2024 Jan 18;14(1):94. doi: 10.3390/brainsci14010094 (PMC10813864; doi:10.3390/brainsci14010094)
Supplement: Supplementary file 1 [file brainsci-14-00094-s001.zip › brainsci-2823493-supplementary.pdf]

Supplemental Material

Table S1. Results of linear regression analyses for regional OEF and rCBF, with sex as an additional covariate.

Model	Dependent variable	Independent variables	Coefficient ± Standard Error	*P*-value
OEF1	OEF in frontal SSS	Group	5.17 ± 2.54 %	0.054
		Age	0.11 ± 0.051 %/year	0.04
		Sex	-0.59 ± 2.31 %	0.80
OEF2	OEF in posterior SSS	Group	2.92 ± 2.67 %	0.29
		Age	0.075 ± 0.053 %/year	0.17
		Sex	-2.10 ± 2.43 %	0.40
OEF3	OEF in ICV	Group	5.20 ± 3.87 %	0.20
		Age	0.074 ± 0.078 %/year	0.35
		Sex	4.74 ± 3.53 %	0.19
**Model**	**Dependent variable**	**Independent variables**	**Coefficient ± Standard Error**	***P*-value**
rCBF1	rCBF in frontal gray matter	Group	-0.085 ± 0.025	0.003
		Age	-0.0018 ± 0.00050 /year	0.002
		Sex	-0.026 ± 0.022	0.28
rCBF2	rCBF in gray matter of all brain lobes	Group	-0.018 ± 0.015	0.25
		Age	-0.00056 ± 0.00030 /year	0.08
		Sex	-0.00079 ± 0.014	0.96
rCBF3	rCBF in BGT	Group	0.024 ± 0.031	0.45
		Age	0.0030 ± 0.00062 /year	0.0001
		Sex	-0.043 ± 0.028	0.14

Group = 1 for COVID-19 ICU survivors and Group = 0 for healthy controls.

Abbreviations: OEF: oxygen extraction fraction; rCBF: relative cerebral blood flow, normalized by global CBF; SSS: superior sagittal sinus; ICV: internal cerebral veins; COVID-19: coronavirus disease 2019; ICU: intensive care unit.
